# Bismuth Vanadate Decked Polyaniline Polymeric Nanocomposites: The Robust Photocatalytic Destruction of Microbial and Chemical Toxicants

**DOI:** 10.3390/ma16093314

**Published:** 2023-04-23

**Authors:** Jari S. Algethami, M. Shamshi Hassan, Touseef Amna, Laila S. Alqarni, Mohsen A. M. Alhamami, Amal F. Seliem

**Affiliations:** 1 Department of Chemistry, College of Science and Arts, Najran University, Najran 11001, Saudi Arabia; 2 Promising Centre for Sensors and Electronic Devices (PCSED), Advanced Materials and Nano-Research Centre, Najran University, Najran 11001, Saudi Arabia; 3 Department of Chemistry, College of Science, Albaha University, Albaha 65799, Saudi Arabia; 4 Department of Biology, College of Science, Albaha University, Albaha 65799, Saudi Arabia; 5 Chemistry Department, College of Science, Imam Mohammad Ibn Saud Islamic University (IMSIU), Riyadh 11564, Saudi Arabia

**Keywords:** BiVO_4_@PANI, environmental remediation, photocatalyst, antibacterial, nanocomposite

## Abstract

Functional materials have long been studied for a variety of environmental applications, resource rescue, and many other conceivable applications. The present study reports on the synthesis of bismuth vanadate (BiVO_4_) integrated polyaniline (PANI) using the hydrothermal method. The topology of BiVO_4_ decked PANI catalysts was investigated by SEM and TEM. XRD, EDX, FT-IR, and antibacterial testing were used to examine the physicochemical and antibacterial properties of the samples, respectively. Microscopic images revealed that BiVO_4_@PANI are comprised of BiVO_4_ hollow cages made up of nanobeads that are uniformly dispersed across PANI tubes. The PL results confirm that the composite has the lowest electron-hole recombination compared to others samples. BiVO_4_@PANI composite photocatalysts demonstrated the maximum degradation efficiency compared to pure BiVO_4_ and PANI for rhodamine B dye. The probable antimicrobial and photocatalytic mechanisms of the BiVO_4_@PANI photocatalyst were proposed. The enhanced antibacterial and photocatalytic activity could be attributed to the high surface area and combined impact of PANI and BiVO_4_, which promoted the migration efficiency of photo-generated electron holes. These findings open up ways for the potential use of BiVO_4_@PANI in industries, environmental remediation, pharmaceutical and medical sectors. Nevertheless, biocompatibility for human tissues should be thoroughly examined to lead to future improvements in photocatalytic performance and increase antibacterial efficacy.

## 1. Introduction

The water contamination has been a major issue, since the turn of the century, with substantial consequences on both the natural environment and human existence [1,2]. Organic dyes, benzene-based organics, and phenols are the primary contaminants of concern in wastewater because of their elevated toxicities and inability to degrade [3]. Similarly, bacterial infections and the growth of multidrug-resistant bacteria can both be lethal. As a result, improved antibacterial materials and constructs are of tremendous interest. In this regard, metals and their ions have been utilized to reduce the risk of bacterial infection for decades, and more recently, metal-based nanomaterials with better antibacterial characteristics have been recommended as novel and configurable options [4]. Around 70% of bacterial infections are known to be resistant to one or more antibiotics commonly used to treat infections. Infectious bacterial diseases are a major public health and economic concern [5]. Antibiotics have three predominant bacterial targets; cell wall synthesis, DNA replication, and the protein-translational machinery [6]. With the prevalence of antibiotic-resistant bacteria, effective and long-term antibacterial materials are desperately needed. Therefore, the development of novel and potent antibacterial drugs is critical [7,8,9]. Metals have been utilized for many years and extensively researched for their antibacterial properties. The antimicrobial action of Ag, Cu, Au, Ti, Zn, and other metals, each with different characteristics, potencies, and activity spectrums, have been identified and exploited for centuries [10,11,12]. Lately, nanotechnology has opened up new opportunities in many science and technology domains. Researchers are becoming increasingly interested in pharmaceutical nanotechnology due to its multiple benefits [13]. As a result, finding effective, green, and non-toxic purification technologies and materials capable of efficiently removing organic pollutants from wastewater is critical for environmental protection [3].

Furthermore, the application of synthetic dyes, which are among the most hazardous pollutants, is expanding due to a growing need for them across several industrial sectors, including paper, cosmetics, textile, leather, and food industries. Due to increased wastewater output from the dye industry, which also interferes with photosynthesis and the biological cycle and devastates the ecosystem of natural water bodies, there is an increase in aesthetic pollution [14]. The alleged contaminant that has been heavily utilized in the textile sector is rhodamine B dye. Numerous other items, including ballpoint pens, paints, leather, dye lasers, carbon sheets, stamp pad inks, crackers, and explosives are also produced using this dye [15]. Rhodamine B dye is a relatively weak basic nitrogenous molecule that undergoes disintegration and produces highly stable and non-biodegradable colorful cations. These vibrant cations infiltrate surface and groundwater reservoirs and significantly affect the aquatic ecology. It is widely present in the aquatic environment, which could be harmful to both human and animal health as it can result in mutagenic and carcinogenic changes [16]. It is also classified as a neurotoxic dye and can affect the eyes, skin, digestive and respiratory systems [16]. 

Photocatalysis is an environmentally friendly method of treating wastewater and is also an alternative source of renewable energy. Industrial water is vulnerable to contamination from organic pigments and dyes that have detrimental environmental effects [17]. In photocatalysis, the electrons are stimulated from the valence band to the conduction band upon the absorption of photons with energy greater than the photocatalyst’s band gap, forming electron holes. To start a series of photocatalytic events, these charge carriers either combine again or move to the surface. When a semiconductor is exposed to visible light, a photocatalytic process that produces a large number of reactive radicals is a more environmentally friendly way to destroy organic contaminants than the current methods used [17,18]. Visible-light photocatalysts have recently gained considerable interest because of their effective use of sunlight, ease of reusing, and easy chemical synthesis. One of the novel visible-light-driven photocatalysts is bismuth vanadate (BiVO_4_), which comes in three different crystalline forms. The crystal shape of BiVO_4_ has a significant impact on both its photophysical and photocatalytic capabilities. When compared to tetragonal BiVO_4_, monoclinic BiVO_4_ exhibits higher photocatalytic activity. Due to its narrow band gap, monoclinic BiVO_4_ has been identified as a potentially appropriate visible light photocatalyst for the degradation of organic contaminants [19,20] as well as for O_2_ evolution when exposed to sunshine. BiVO_4_ has a low-cost production and is non-toxic in nature. Its increased use as a reliable photocatalytic material is primarily due to its stable crystal structure, high light quantum and electronic transmission efficiency, and exceptional energy usage capacity. Unfortunately, the quick recombination of photoinduced electrons and holes greatly restricts the practical applications of pure BiVO_4_ in photocatalytic pollutant degradation, rendering the photocatalytic activity of pure BiVO_4_ often insufficient. Consequently, a crucial problem is how the separation efficiency of photo-generated electrons and holes could be intensified to boost the visible-light photocatalytic performance of BiVO_4_ photocatalysts [19,20,21,22]. It has long been known that improving the separation effectiveness of photoinduced electron-hole pairs can enhance the photocatalytic activity of the photocatalysts [23]. 

Currently, polymer-metal oxide composites have been under consideration due to their many potential uses. One of the most popular polymers for creating nanocomposites is polyaniline (PANI), which has high conductivity, strong environmental durability, and distinctive electron and hole transportation capabilities [24]. PANI is a celebrated conducting polymer that is frequently investigated because of its attractive properties viz., photocatalytic, antibacterial, biocompatibility, and easy synthesis method. PANI can be modified through protonation with various dopant acids, altering its structure and characteristics. However, the application of PANI becomes restricted due to its low processability and insolubility [25,26]. By mixing various materials or metal ions, novel composites with new applications can be produced [27]. In general, nanocomposites based on organic polymers have many advantages, such as long-term stability, good processability, and outstanding optical, catalytic, electronic, and magnetic properties. Therefore, resultant nanocomposites could potentially provide many applications [28]. PANI-based nanocomposites have high reduction and oxidation potential that can be explored for simultaneous H_2_ generation and degradation of water pollutants. The combination of the photocatalyst and PANI appears to be appropriate for boosting photoactivity under visible light, given the effective carrier-transfer property of PANI [21]. Only limited research has examined the effects of combining PANI with straightforward metal oxide catalysts to enhance photocatalytic activity [21,29]. For the study’s applicability to be expanded, more research is required. Bismuth Vanadate has been identified as a photocatalyst for water splitting [30] and the eradication of dye pollutants, with a high degree of mineralization under visible-light irradiation [19,22]. 

There are some interesting studies about the synthesis of polymeric nanocomposites in the literature which demonstrate efficient antibacterial action. The antimicrobial activities of nanocomposites, for instance, as synthesized with Ag/polyvinylpyrrolidone, were tested against various bacterial and fungal strains and a strong antimicrobial property was discovered [31]. Recently, researchers have applied various strategies to improve antibacterial and antibiofilm properties, including the direct modification of membrane material [24,32], or blending or coating with bioactive or chemical moieties having antibacterial properties such as metal ions or nanoparticles (NPs) [33,34]. A few previous investigations have also reported the antibacterial action of similar composites, such as Bi_2_WO_6_/Ag [35] and BWO/GO [36] due to photogenerated oxyradicals, which are responsible for the destruction of the bacterial structure. Very recently, CNFs-Bi_2_WO_6_ nanocomposites were also reported to possess potent antimicrobial activity against pathogenic bacteria as well as fungal strains [37]. Considering the versatility of PANI and BiVO_4_ in this study, the synthesis and detailed characterization of BiVO_4_-decked PANI catalysts was carried out and these nanocomposites were evaluated as photocatalysts for dye degradation and antimicrobial activity. The photocatalytic activity has been studied for the degradation of rhodamine B (RhB) under visible light, and the antimicrobial activity was investigated using *E. coli* as a model strain. It was expected that the introduction of PANI would not only enhance the light absorption of the nanocomposite but would also ease the parting of the photogenerated electron-hole pairs, together resulting in enhanced photocatalytic activity and antibacterial activity. This nanocomposite can practically be applied to purify contaminated water and control waterborne diseases. The design and synthesis of this functional composite (BiVO_4_-decked PANI) holds significant promise for future applications and the development of next-generation remedies to current therapeutic and environmental problems. Henceforth, this polymeric nanocomposite will serve as a functional material for the robust photocatalytic destruction of microbial and chemical toxicants and aid in environment-cleaning applications. 

## 2. Materials and Methods

Aniline and ammonium persulfate (APS) were purchased from Sigma-Aldrich, St. Louis, MO, USA. The Japanese company Showa Chemicals Ltd, Tokyo, Japan, provided citric acid and N, N-dimethylformamide (DMF, 99.5 test). The ammonium metavanadate (>99.5%) and bismuth nitrate pentahydrate (>99.5%) were supplied by Kanto Chemical in Tokyo, Japan. No extra purification was required before use because each reagent was of analytical-grade purity.

### 2.1. The Synthesis of the PANI Nanotube (NTs)

To make the blend solution, 500 mL of distilled water and 4.0 g of citric acid were combined with 0.1 M of aniline. The 0.1 M APS aqueous solution was added and stirred concurrently. The sample was then maintained in a refrigerator at 4 °C for 24 h. The result was a dark green suspension. The suspension was filtered, cleaned with distilled water and ethanol to remove remnants, and dried overnight at 60 °C under the vacuum.

### 2.2. The Synthesis of the BiVO_4_@PANI Nanocomposite

The hydrothermal preparation procedure was used for the preparation of the BiVO_4_@PANI composite. Ammonia solution (20 mL) was mixed with ammonium metavanadate (1 mM) and agitated for 20 min. Separately, a clear solution was obtained by stirring Bi(NO_3_)_3_·5H_2_O (1 mM) in ethanol and acetic acid in a 1:1.5 ratio. The combination of both solutions gradually formed a homogeneous orange–yellow mixture. PANI NTs (35 mg) were then added to the completed solution while constantly stirring. The resulting solution was exposed to a 20 h hydrothermal autoclave treatment at 120 °C without any pH adjustments. The precipitate was rigorously washed with distilled water and dehydrated at 80 °C. The same procedure as described above was utilized to produce pure BiVO_4_ without PANI.

### 2.3. Characterization

The crystal structure of the synthesized product was determined using X-ray diffraction (XRD, Rigaku, Tokyo, Japan) with Cu KR radiation (=1.5418) at a scan rate of 6° per minute and an angle range of 10 to 70°. To examine the microstructure and crystalline pattern, images of samples were taken using transmission electron microscopy (TEM, H-7650, Hitachi, Tokyo, Japan). The chemical conformations of the samples were scrutinized by an Energy dispersive X-ray (EDX) spectrometer. The spectrometer acquired Fourier transform infrared (FTIR) spectra using the KBr pellet technique (Varian FTS 1000). The light absorption of the samples was measured via a UV-Vis diffused reflectance spectrum (UV-DRS, 525 Shimadzu, Tokyo, Japan). To obtain morphological information, the samples were homogeneously spurted on carbon tape, Os metal coating was carried out for 10 s and the pictures were attained at different resolutions using field emission scanning electron microscopy (FE-SEM, JEOL JSM6700, Tokyo, Japan).

### 2.4. Photocatalytic Activity

To test the photocatalytic action of PANI, BiVO_4_, and BiVO_4_@PANI, 250 mg of the sample was pooled in 250 mL of 10 ppm Rhodamine B (RhB) aqueous solution and continuously stirred. The experiments were carried out at room temperature, and the slurry was aerated for 30 min in the dark to achieve dye adsorption–desorption equilibrium on the sample’s surface before being exposed to visible light (>400 nm). At regular intervals, 3 mL aliquots of the suspension were removed and immediately centrifuged at 12,000 rpm. The absorbance of the RhB solution was certified using a UV-Vis spectrophotometer (Shimadzu UV-3101).

### 2.5. Antibacterial Activity

Pure culture from Trypticase Soy Agar (TSA) plates was grown into Tryptic Soy Broth (TSB) and incubated overnight to examine the bactericidal capabilities of PANI, BiVO_4_, and BiVO_4_@PANI. The overnight culture was immersed in 1 mL of fresh TSB broth in 100 mL of freshly produced TSB and incubated in a rotary shaker at 37 °C with 150 rpm. A UV-visible spectrophotometer (UV-2550, Shimadzu, Kyoto, Japan) was used to measure the optical density (OD) at 600 nm (10^8^ CFU/mL). After that, 1 mL of previously reported culture inoculum was added to the freshly made TSB enriched with plain PANI, BiVO_4_, and BiVO_4_@PANI nanocomposites (0–400 µg/mL). The OD assessment indicated above was used to measure the rate of increase at 4 h increments for 16 h. Some bacterial broth suspensions were not exposed to PANI, BiVO4, and BiVO_4_@PANI nanocomposites as a control.

## 3. Results and Discussion

Clean and renewable energy, through visible-light-driven photocatalysis, for environmental pollution management is of great interest [23,38]. In comparison to conservative approaches, photocatalysis demonstrates better potential in environmental remediation, owing to price, absence of detrimental derivatives, and durable oxidative competence for pollutants under conventional conditions [39,40,41]. The capacity to integrate the benefits from several facets to act in symphony to accomplish set targets makes composite materials very appealing. 

**Figure 1:**
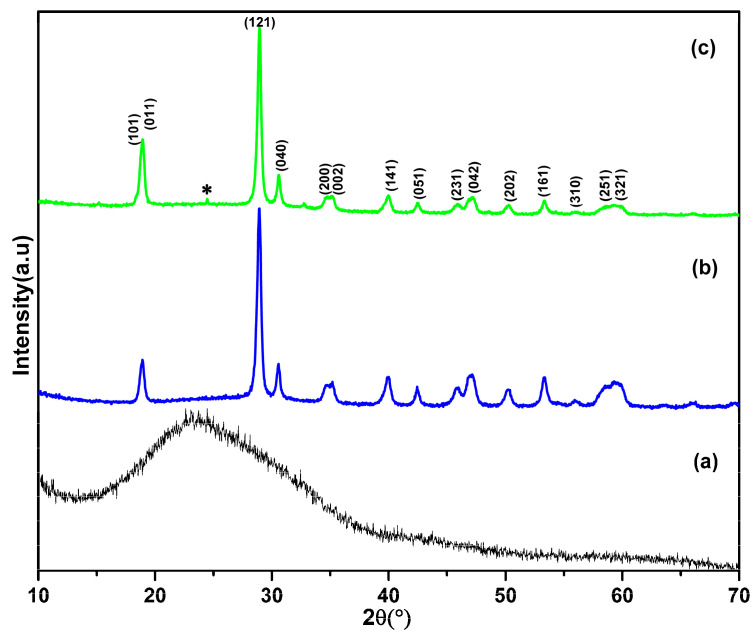
The XRD pattern of (**a**) pure PANI, (**b**) BiVO_4_, and (**c**) BiVO_4_-PANI nanocomposite. “*” represents an unidentified peak.

The BiVO4@PANI composite was synthesized in the present study using the hydrothermal approach. As demonstrated in Figure 1, X-ray diffraction (XRD) was utilized to inspect the crystalline stage of pure PANI, BiVO_4_, and BiVO_4_@PANI nanocomposites. The amorphous form of PANI is indicated by a broad diffraction peak between 15 to 30 degrees, having a maximum intensity of around 25 degrees (Figure 1a). It represents the distinctive peak of the PANI and thus supports the data presented in the literature [42,43]. According to the XRD spectra of the BiVO_4_ sample (Figure 1b), the acquired diffraction peaks may be attributed to the pristine monoclinic stage of BiVO_4_ with JCPDS Card No. 14-0688 [19,44], and no additional adulterations have been identified. The XRD analysis of the BiVO_4_@PANI composite reveals strong peaks that are compatible with pure monoclinic scheelite BiVO_4_. It was discovered that PANI does not affect the crystallinity of BiVO_4_. An insignificant shoulder peak in BiVO_4_@PANI composite sample around 24 degrees could not be identified, perhaps due to some noise or impurity. There are no identifiable diffraction peaks in the spectra that correspond to those of PANI, indicating that the PANI in the composites occurs in an amorphous arrangement (Figure 1c). 

**Figure 2:**
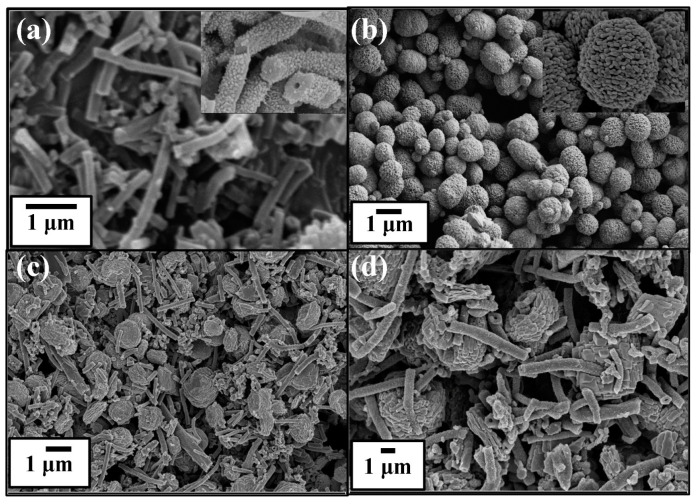
The FESEM images of (**a**) pure PANI (inset shows high magnification), (**b**) BiVO_4_ (inset shows high magnification), and (**c**,**d**) BiVO_4_@PANI nanocomposite at different magnifications.

The morphology of pure PANI, BiVO_4_, and BiVO_4_@PANI nanocomposites is investigated by SEM and is shown in Figure 2. Pure PANI seems to be a tube-like structure. Synthesized PANI NTs have a diameter of 100 to 200 nm and are a few micrometers (Figure 2a). At high resolution, images of PANI showed cylindrical tube-like structures with a granular surface (inset Figure 2a). From the SEM image (Figure 2b), the produced BiVO_4_ sample spears as homogeneous hollow cage assemblies, where the cage is a collection of tiny nano-beads. The nano-beads possess typical lengths and widths of 100 and 50 nm, respectively (inset Figure 2b). On the other hand, the BiVO_4_@PANI nanocomposite shows a homogeneous blend of the NTs and nanocage structure (Figure 2c,d). Usually, the photocatalysts with hollow structures like NTs and nanocages show higher specific surface areas, which affords a plan for the adsorption of organic molecules and leads to improvements in photocatalytic efficiency. 

**Figure 3:**
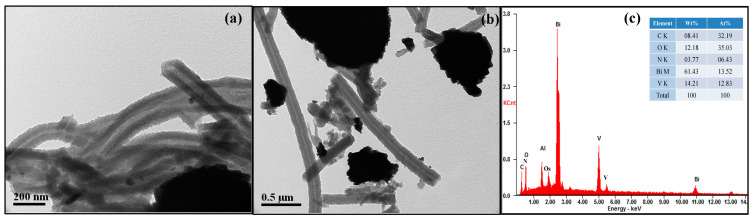
(**a**,**b**) TEM images and (**c**) EDX pattern of BiVO_4_@PANI composite.

The structure of the produced composite was validated once more by TEM imaging. The PANI NTs with BiVO_4_ cage structure are shown in Figure 3a,b. The cage image of BiVO_4_ is dark and opaque because of the thickness of the sample. This signifies that the TEM picture has no depth sensitivity. The presence of elements in the composite BiVO_4_@PANI was further examined using EDX analysis (Figure 3c). The EDX revealed the peaks of C, V, Bi, O, and N with atomic and weight percentages (%), respectively. Thus, the EDX also confirms the formation of a nanocomposite comprised of BiVO_4_ and PANI alone.

**Figure 4:**
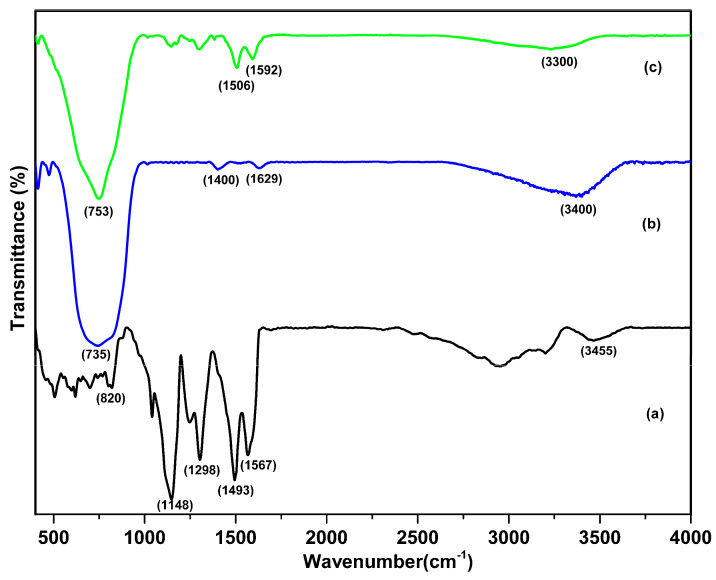
The FTIR spectra of (**a**) pure PANI, (**b**) BiVO4, and (**c**) BiVO_4_@PANI composite.

Figure 4a depicts a typical FTIR of pure polyaniline. It shows major distinctive peaks at 1561 cm^−1^ and 1493 cm^−1^, which were attributed to C=N stretching of the quinoid and C=C stretching of the benzoid rings, respectively. The bands at 1240 and 1298 cm^−1^ correspond to C-N stretching modes in the benzene and quinone rings, respectively. The stretching vibration peak of the quinone ring is shown by the absorption peak at approximately 1148 cm^−1^. The N-H bond stretching vibration peak of PANI is located at about 3450 cm^−1^. The absorption peak at 820 cm^−1^ is the C-H bond-bending vibration peak of the disubstituted benzene ring’s outer surface [45,46]. The IR spectra of the synthesized BiVO_4_ sample are shown in Figure 4b. A significant bandwidth was seen between 600 and 800 cm^−1^ due to V-O stretching (Figure 4b,c) [19]. Pure BiVO_4_ and the composite have a broad peak centered around 3400 and 3300 cm^−1^ which is attributed to the O-H stretching vibration of the water molecules from the environment. When compared to PANI, the IR bands in the nanocomposites have favorably moved to higher wavenumbers (Figure 4c). These alterations in the specific bands are brought on by interactions between PANI and BiVO_4_. This association may result from the oxygen atoms on the surface of the transition metal oxides forming hydrogen bonds with the N-H of PANI, which binds oxide particles to PANI chains. The σ–п bond interaction between the metal oxide and polyaniline involves the ᴨ molecular orbital of PANI overlapping the vacant d-orbital of metal ions to form the σ-bond. Further, the п* molecular orbital of PANI overlapping the d-orbital of metal ions to form ᴨ-bond also facilitates the attachment of metal particles to the polymer chains [47].

**Figure 5:**
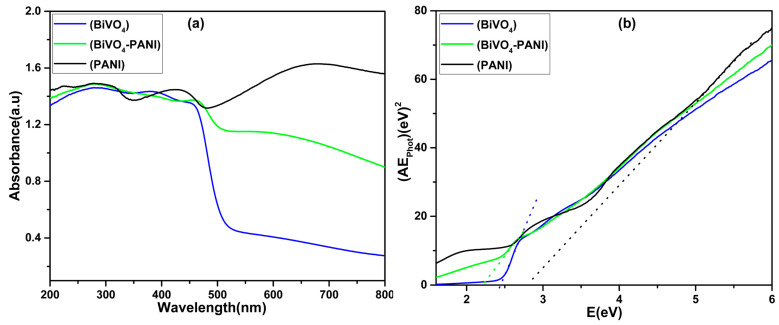
(**a**) The UV-Vis diffuse reflectance spectra and (**b**) plot of Kubelka–Munk function vs. photon energy of the pure PANI, BiVO_4_, and BiVO_4_@PANI nanocomposite. Black, blue and light green dotted lines represent the extrapolation of graphs of PANI, BiVO_4_, and BiVO_4_@PANI nanocomposite respectively.

The optical absorption properties of photocatalysts are critical in the photocatalysis process. The UV-vis spectra of pure PANI, BiVO_4_, and composite BiVO_4_@PANI are shown in Figure 5A. The distinctive peaks of PANI appear at around 290, 430, and 710 nm. These are attributed to the π − π*, doping level, and the production of polarons (quinoid segments), respectively, which indicate the existence of PANI molecules. The unalloyed PANI can absorb not only UV light but visible light and near-infrared areas in addition, which can be attributed to transitions in the PANI molecules. The photoabsorption of the BiVO_4_ sample ranges from UV to visible light. The spectra of the BiVO_4_@PANI composite demonstrate greater absorption towards the visible light area than pure BiVO_4_, meaning that the BiVO_4_@PANI composite can boost the photo-response to the visible light range. As a result, we can conclude that the BiVO_4_@PANI composite absorbs more photons and generates additional electron-hole pairs under the same visible light irradiation, resulting in increased photocatalytic activity. The band gap energy (E_g_) of all three samples can be calculated from the graph of (Ahν)^2^ versus hν using the equation, Ahν = (hν − Eg)^n/2^ [48] (Figure 5B). The E_g_ for PANI, BiVO_4_, and BiVO_4_-PANI is found to be 2.82, 2.44, and 2.22 eV. The calculated E_g_ for PANI and BiVO_4_ is per the reports in the literature [19,49]. 

**Figure 6:**
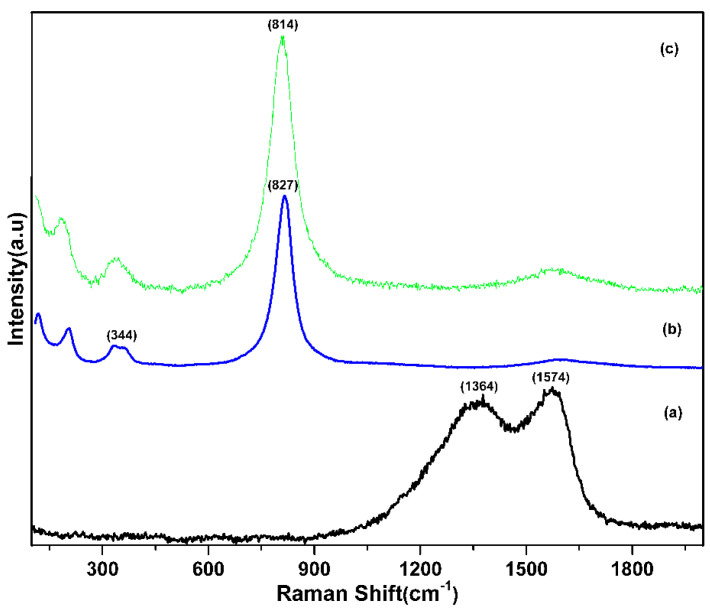
The Raman spectra of the (**a**) pure PANI, (**b**) BiVO_4_, and (**c**) BiVO_4_@PANI nanocomposite.

Raman spectroscopy is established as a valuable technique for the characterization of the structure of various materials. Figure 6 depicts the Raman spectra of pure PANI, BiVO_4_, and the composite BiVO_4_@PANI. The virgin PANI Raman spectra have two large peaks at ~1364 and ~1574 cm^−1^ (Figure 6a). The presence of νC–N of Polaron (where ‘ν’ signifies a transitional bond between a single and a double) stretching mode of the delocalized polaronic charge carriers in PANI is connected with the occurrence of the broad Raman band at 1364 cm^−1^. Furthermore, the Raman bands at 1574 cm^−1^ are linked to the C=C stretching of the quinoid rings [42]. The band at 827 cm^−1^ in the Raman spectra of BiVO_4_ related to the stretching of the increased V-O bond length demonstrates the improvement in the crystalline nature [50]. The (VO)_4_^3−^ modes are represented by a weak shoulder peak at 344 cm^−1^ (Figure 6b) [20]. Furthermore, the Raman spectrum of the BiVO_4_@PANI composite demonstrates that the inclusion of PANI does not affect the absorption bands when compared to pure BiVO_4_. In the instance of BiVO_4_@PANI, a change of the Raman band to a lower wavenumber, from 827 to 814 cm^−1^ attributed to the symmetric V-O bond stretching mode, demonstrates that the VO_4_ tetrahedral’s average lone-range symmetry becomes less regular (Figure 6c). The blue shift is caused by the charge transfer and contact between BiVO_4_ and PANI, which causes interaction and surface strain. As a result, the structure was validated by the Raman spectra of the synthesized materials.

**Figure 7:**
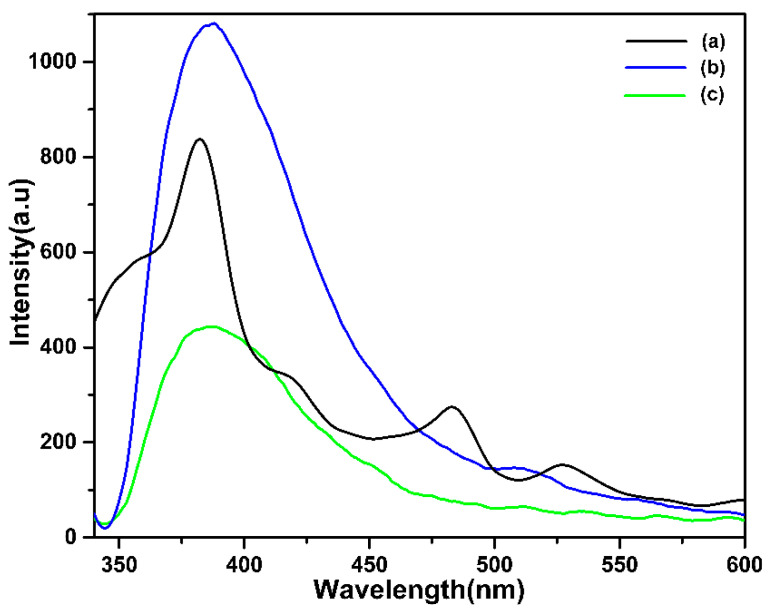
The PL spectra of the (**a**) PANI, (**b**) BiVO_4_, and (**c**) BiVO_4_ @PANI nanocomposite.

The photoluminescence experiment is used to assess the efficiency of charge carrier entrapment, transport, and separation of the photogenerated electrons and holes in semiconductors. We know that effective electron-hole pair separation is an important component that influences composite photocatalyst performance [51]. Figure 7 depicts the PL spectra of pure PANI, BiVO_4_, and the composite BiVO_4_@PANI. According to the graph, the BiVO_4_@PANI composite has the lowest PL intensity among PANI and BiVO_4_ (Figure 7c). It means that, when exposed to visible light, the PL of a composite demonstrates a decreased rate of electron and hole recombination. This is principally due to charge transfers at the PANI and BiVO_4_ heterojunction contacts, which separate electrons and holes.

The use of multiple photocatalyst components in heterojunctions is a promising way to increase photocatalytic activity through improved charge carrier separation and decreased recombination. The efficiency of photocatalysis can be significantly increased by carefully selecting the respective band locations in semiconductor composites made of two distinct materials. So, creating efficient strategies for enhancing charge separation efficiency and expanding the spectral response range is essential. These problems might be resolved by creating a heterojunction nanostructure between a photocatalyst and a small band gap semiconductor with matched band potentials. Several studies have been conducted that demonstrate how PANI nanocomposites are created and how active they are as photocatalysts. (Table 1). 

**Figure 8:**
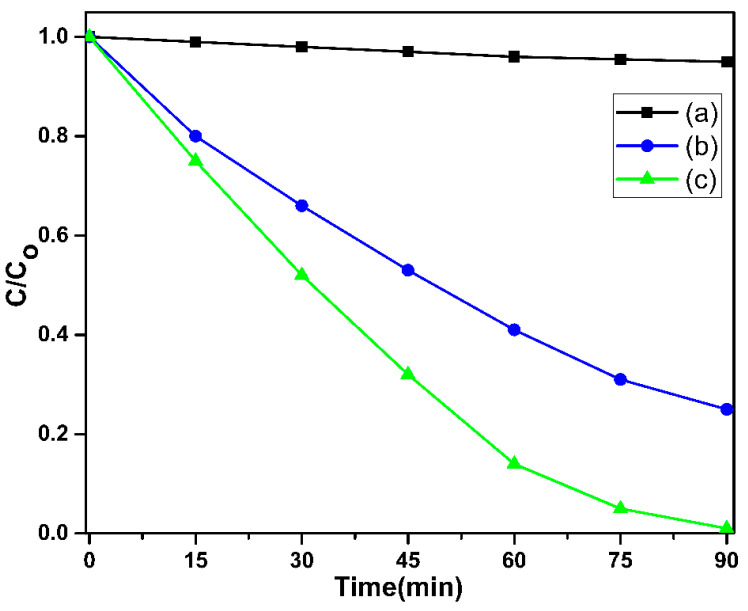
The photocatalytic degradation of the RhB dye solution by (**a**) pure PANI, (**b**) BiVO_4_, and (**c**) BiVO_4_@PANI nanocomposite.

In the present study, the photodegradation of RhB was measured under visible light irradiation (>400 nm) to test the efficacy of PANI, BiVO_4_, and BiVO_4_@PANI photocatalysts. After 90 min of reaction, pure PANI and BiVO_4_ have degradation efficiencies of 5% and 60%, respectively (Figure 8a,b). Other researchers have observed similar findings [19,42,52], whereas BiVO_4_@PANI composite photocatalysts demonstrated 100% efficiency in the same period (Figure 8c). Therefore, the interaction between BiVO_4_ and PANI improves the photocatalytic activity of the composite photocatalyst.

**Figure 9:**
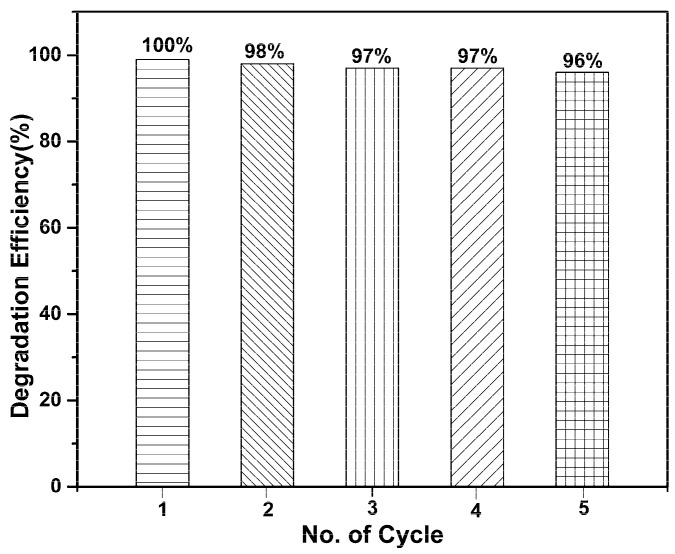
The photocatalytic efficiency of RhB dye solution by BiVO_4_@PANI nanocomposite in cycles.

Recycling stability as well as a strong photocatalytic activity are critical concerns for the long-term use of catalysts in practical applications. During repeatability investigations, the stability of RhB degradation on BiVO_4_@PANI was investigated. The photocatalytic effectiveness remained at 96% even after five cycles, demonstrating that the BiVO_4_@PANI composite is highly stable under visible light (Figure 9).

**Figure 10:**
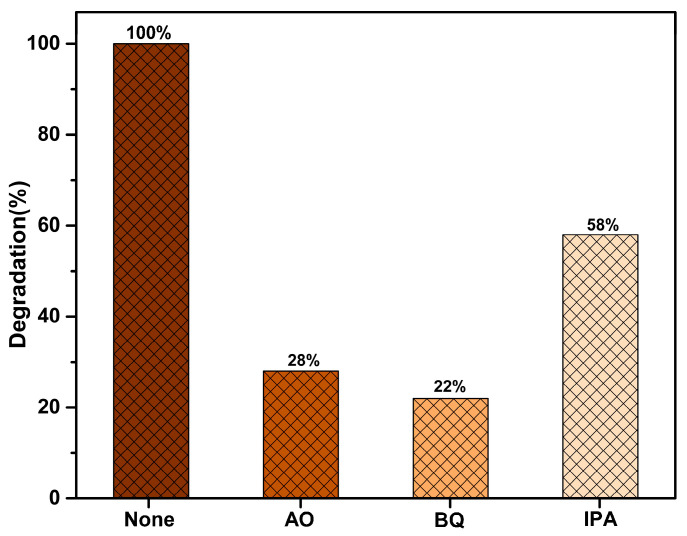
Trapping experiments of active species in the photocatalytic reaction.

Scavenging studies are most effective to comprehend the reaction process and identify the key active species. Similar to previous tests (degradation), a new radical scavenger (1 mmol) was added to the photocatalytic reaction solution and exposed to visible light for 90 min, as shown in Figure 10. Trapping studies using isopropanol (IPA), ammonium oxalate (AO), and benzoquinone (BQ) as scavengers to quench ^•^OH, h^+^ and ^•^O^−^_2_, respectively, were carried out to determine the principal reactive species generated in the current system. As shown in Figure 10, 100% photodegradation of RhB over the BiVO_4_@PANI nanocomposite photocatalyst is depicted without the use of scavenging agents. In the presence of IPA, the elimination rate of RhB dropped to 58%. When AO and BQ were present, the degradation rate subsequently dropped to ~28% and 22%, respectively. The outcome was that the primary species involved in the photodegradation of RhB are ^•^OH, h^+^ and ^•^O^−^_2_.

**Scheme 1:**
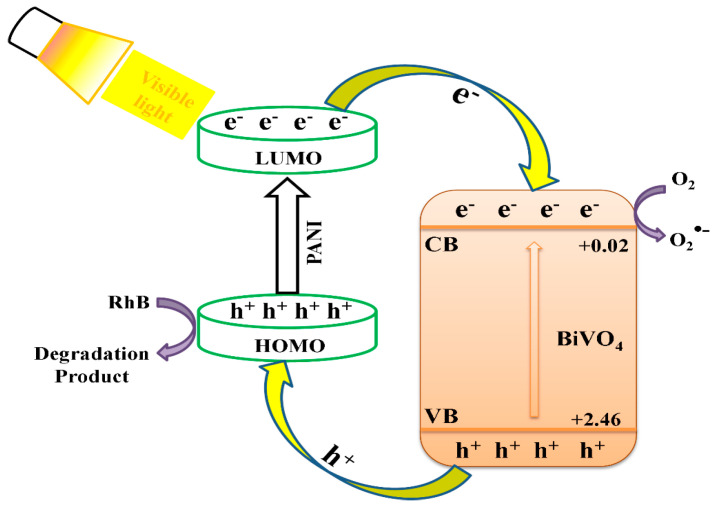
A band structure diagram and electron-hole separation of BiVO_4_@PANI photocatalyst.

Scheme 1 depicts a diagrammatic representation of the mechanism of the charge separation and photocatalytic procedure done by the BiVO_4_@PANI photocatalyst. The conduction band (CB) position of BiVO_4_ was lower than the LUMO position of PANI, so it acts as a sink for the photogenerated electrons in the composite photocatalysts. The valence band (VB) position of BiVO_4_ was lower than the HOMO position of PANI, so the latter can serve as an acceptor for the photogenerated holes in the crossbreed photocatalysts [53,54]. Visible light can excite both BiVO_4_ and PANI, resulting in photogenerated carriers and excited electrons, respectively. PANI-excited state electrons can easily inject into BiVO_4_ CB. At the same time, the photogenerated holes in the VB of BiVO_4_ can freely travel to the surface of the composite via the HOMO of PANI. As a result, photogenerated electrons and holes flow in opposite directions, lowering the likelihood of recombination and increasing charge separation efficiency, which results in enhanced photocatalytic activity. Meanwhile, photogenerated holes rapidly transferring to solution successfully enhanced photo corrosion inhibition. Consequently, the role of PANI in the proposed BiVO_4_@PANI composite photocatalysts can be shown by injecting electrons into CB of BiVO_4_ under visible light irradiation, causing the generation of superoxide radical ions ^•^O^−^_2_ and hydroxyl radical ^•^OH. Ultimately, the ^•^OH species are capable of degrading the dye (RhB) into harmless products [55,56].

**Table 1:** A few documented studies about polyaniline polymeric nanocomposites and their use in photocatalysis and as antimicrobial agents.

Polyaniline Polymeric Nanocomposites	Application	Reference
PANI/TiO_2_ nanocomposite	Photocatalysis	[57]
PANI/CdO nanocomposite	Photocatalysis	[58]
PANI/ZnO nanocomposite	Photocatalysis	[59]
Fe_3_O_4_ @PANI/TiO_2_	Photocatalysis	[60]
Al-doped ZnO-PANI	Photocatalysis	[61]
PANI/PVA (polyvinyl alcohol)/Ag	Antimicrobial	[62]
PANI@ZnO	Antimicrobial	[63]
PANI/Pt-Pd	Antimicrobial	[64]
PANI/Ag–Pt	Antibacterial	[65]
PANI-Ag-Au	Antibacterial	[66]
PANI-Zn@CuO	Antibacterial	[67]
BiVO_4_@PANI nanocomposite	Antimicrobial and Photocatalysis	This study

**Figure 11:**
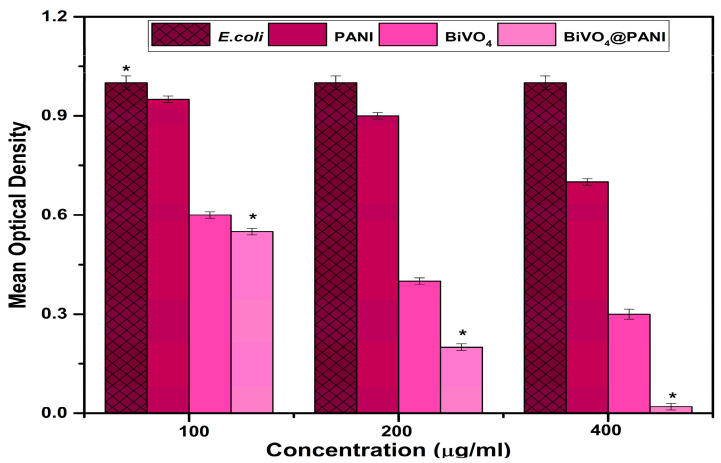
*E. coli* sensitivity to pure PANI, BiVO_4_, and BiVO_4_@PANI nanocomposites (0–400 µg/mL). The data are presented as the mean and standard deviations of three replicates. * p < 0.005 vs. control.

Pure PANI, BiVO_4_, and BiVO_4_@PANI nanocomposites were tested for antimicrobial properties. The growth pattern of bacteria cultivated on pure PANI was considerably slower at a higher concentration than that of pristine bacteria, signifying the antibacterial effectiveness of PANI. BiVO_4_ nanocages, on the other hand, showed a significant reduction in bacterial count. BiVO_4_ cages added to the PANI matrix improved the antibacterial activity of the BiVO_4_@PANI NTs significantly (Figure 11)., The bactericidal growth rate was also drastically reduced, which could be related to variations in architecture and nanocomposite features. The data show that nanocomposites have a high potential for removing biological and chemical pollutants. 

**Scheme 2:**
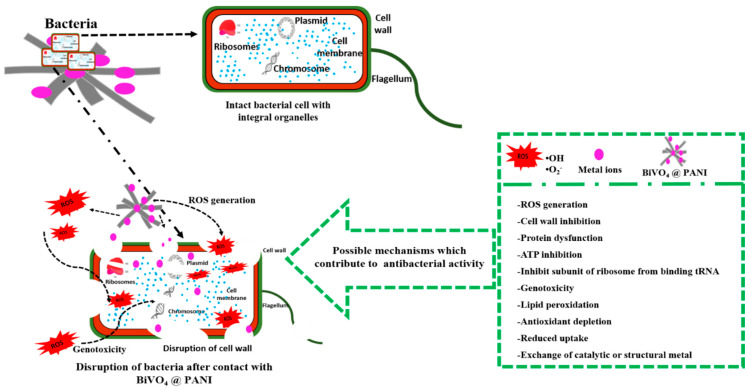
A schematic illustration of the possible antibacterial mechanisms of synthesized BiVO_4_@PANI, which contributed to antibacterial activity.

As expected, the remarkable antibacterial activity could be linked to the generation of free radicals (hydroxyl radicals) (Scheme 2). Bacterial membranes are impaired by these ^•^OH radicals. Possibly these radicals will also infiltrate cells, causing genetic damage, halting the respiratory sequence, and eventually leading to cellular destruction [7,10]. Preceding research investigations (Table 1) have also found that comparable composites, such as Bi_2_WO_6_/Ag [35] and BWO/GO [36], exhibit antibacterial properties due to photogenerated oxyradicals, which are responsible for bacterial structural degradation. Recently, CNFs-Bi_2_WO_6_ nanocomposites were also demonstrated to have substantial antibacterial activity against pathogenic bacteria and fungal strains [37]. Gram-negative outer membranes consist of negatively charged lipopolysaccharides. Phospholipids are major constituents of the bacterial membrane, and they contain reactive phosphoryl groups next to unmodified lipid carboxyl groups, both of which can interact with metal cations at a neutral and alkaline pH. Significantly, the composition of bacterial phospholipids varies between species, and the interaction with metal is mostly determined by the lipid’s outwardly facing polar head group, which is particularly susceptible to perturbations [4,68]. Nevertheless, the metal ions possess various fascinating chemical and physical characteristics, which have a substantial impact on the antibacterial effect of the applied compounds (Table 1). When an ion binds to a membrane, the membrane dipole potential decreases and the hydration of the head group changes. As a result, the total charge of the membrane changes, resulting in a local membrane rupture, increased permeability, and ROS formation [4]. Antibiotics have three predominant bacterial targets: cell wall synthesis, DNA replication, and the protein-translational machinery [6]. The combination of diverse metal ions and nanoparticle surface decorations will result in synergistic effects and microbial death, as well as the ability to reduce potential host adverse effects [4]. Herein, the findings of the present investigation corroborate earlier research studies (Table 1) well. 

## 4. Conclusions

Overall, functional materials have been investigated for a wide range of environmental applications, including water and air purification, resource rescue, and a myriad of other potential uses. The capacity to combine the benefits from several areas to work together to achieve specific goals makes composite materials particularly appealing. In summary, PANI, BiVO_4_, and BiVO_4_@PANI composite were synthesized and thoroughly characterized utilizing a variety of sophisticated physicochemical techniques. PANI, BiVO_4_, and BiVO_4_ decked PANI were tested for antibacterial efficacy against the model organism *E. coli*. SEM was used for topographical comparison, and SEM pictures demonstrated a distinct cage shape of BiVO_4_ and NTs for PANI. Antibacterial and photocatalytic activities were increased due to the elevated surface area and synergism. Polymeric NTs will now be used as a functional material for the powerful photocatalytic eradication of microbiological and chemical toxicants, as well as for environmental cleanup. Nevertheless, before commercialization and implementation in actual applications, these functional materials (PANI, BiVO_4,_ and BiVO_4_@PANI) should be thoroughly tested for human biocompatibility.

## Data Availability

The data presented in this study are available in article.
